# HIV-2 infection as a comparative lens for functional HIV-1 remission

**DOI:** 10.3389/fimmu.2026.1825935

**Published:** 2026-04-17

**Authors:** Sabastine Eugene Arthur, John Atanniba, Prince Adom Nartey, Yeena Abla Tay, Dzidzor Aku Attoh, Christopher Zaab-Yen Abana, Evelyn Yayra Bonney, George Boateng Kyei

**Affiliations:** 1 Medical and Scientific Research Centre, University of Ghana Medical Centre, Accra, Ghana; 2 Department of Virology, Noguchi Memorial Institute for Medical Research, University of Ghana, Accra, Ghana; 3 Departments of Medicine and Molecular Microbiology, Washington University School of Medicine in St. Louis, St. Louis, MO, United States

**Keywords:** block-and-lock, functional cure, HIV-1 remission, HIV-2, immune control, reservoir persistence, SAMHD1, viral latency

## Abstract

Four decades into the HIV pandemic, HIV-2 infection remains an underutilized comparative resource for HIV-1 cure research. HIV-2 is associated with lower plasma viral loads, slower CD4^+^ T-cell decline, and delayed disease progression in many individuals. Early studies attributed these features to intrinsic viral attenuation, pointing to differences in accessory protein function, transcriptional regulation, and reservoir size. However, accumulating molecular and epidemiological evidence challenges this interpretation. The transcriptional status of HIV-2 *in vivo* is not uniform across studies. Some studies report comparable viral RNA levels between HIV-1 and HIV-2 in CD4-matched individuals. In contrast, others find lower per-cell transcriptional output in HIV-2, suggesting that the degree of transcriptional restriction varies with cohort composition, disease stage, and measurement approach. Importantly, neither finding supports a model of uniform proviral silencing. This review examines the molecular biology, immune responses, and reservoir dynamics of HIV-2 infection, weighing evidence that both supports and challenges the view of HIV-2 as an attenuated comparator to HIV-1. The available data suggest that HIV-2 persistence involves regulated viral expression and ongoing, albeit attenuated, immune engagement, rather than transcriptional silence. However, the extent to which immune activation in HIV-2 is quantitatively or qualitatively distinct from that in HIV-1 remains incompletely resolved. HIV-2 does not provide a prescriptive cure blueprint, but it reveals a key biological constraint: ongoing viral transcription can coexist with prolonged immune containment. This finding argues against transcription-only approaches to HIV-1 remission and underscores the need for strategies that combine transcriptional modulation with sustained immune engagement.

## 1 Introduction

Four decades into the HIV pandemic, the pursuit of a functional cure, defined as durable viral suppression in the absence of continuous antiretroviral therapy, has remained largely focused on HIV-1. In contrast, HIV-2, a related lentivirus arising from independent cross-species transmission events, has received comparatively limited attention despite its distinct epidemiological and clinical features. HIV-2 shares major routes of transmission and overall genomic organization with HIV-1, yet differs in transmissibility, plasma viral load dynamics, and rates of disease progression across populations (1). Epidemiological studies from West Africa and Europe have consistently reported lower median plasma viral loads and slower CD4^+^ T-cell decline in many individuals with HIV-2 infection, while also demonstrating that progression to AIDS can occur, particularly in untreated individuals or over extended follow-up periods (2, 3).

These observations have motivated interest in HIV-2 as a comparative model for understanding mechanisms of long-term viral control. Early cohort studies identifying subsets of individuals with relatively preserved CD4^+^ T-cell counts and delayed clinical decline contributed to the perception that HIV-2 infection is associated with a less pathogenic course in many, though not all, individuals (1, 4). While early reports emphasized the attenuated phenotype, longitudinal data have consistently demonstrated that a substantial proportion of untreated individuals ultimately experience immunological decline (2–4). Subsequent virological and immunological investigations have explored whether differences in accessory gene content, host restriction factor interactions, innate immune activation, and adaptive immune responses might contribute to these clinical patterns, including studies on HIV-2’s Vpx interaction with the host restriction factor SAMHD1 and its impact on immune signalling pathways and virus replication dynamics, as well as comparative analyses of immune activation in HIV-1 versus HIV-2 infection (1, 5, 6).

However, it is increasingly clear that many mechanistic inferences drawn from HIV-2 infection are based on indirect evidence and are not uniformly supported across studies. For example, while low or undetectable plasma viremia has often been observed in HIV-2 infection (1), molecular analyses have detected integrated HIV-2 proviral DNA at levels comparable to those seen in HIV-1 infection when similar assays are used (7, 8). It should be noted, however, that this parity in proviral DNA levels has been most consistently demonstrated in individuals with advanced immunosuppression (CD4^+^ T-cell counts below 200 cells/µL) (7, 8); data from aviremic and immunologically preserved individuals suggest that proviral burden may differ across disease stages (9). Likewise, although HIV-2 infection has frequently been discussed in the context of preserved immune function, longitudinal epidemiological studies indicate that a substantial proportion of untreated individuals ultimately experience immunological decline and disease progression (3, 4, 10).

Interpretations that HIV-2 reflects intrinsic viral attenuation have been proposed based on certain clinical and molecular observations, but these interpretations are increasingly viewed as context-dependent rather than broadly generalizable. Studies examining proviral landscapes, transcriptional activity, and inducibility have reported findings comparable to those observed in HIV-1 infection, depending on cohort composition, disease stage, and methodology (1, 7, 8). These discrepancies underscore the heterogeneity of HIV-2 infection and caution against extrapolating conclusions from specific clinical subsets, such as long-term controllers, to the broader infected population.

Accordingly, the premise of this review is to present a comparative biological system for interrogating prevailing assumptions in HIV cure research. By examining where HIV-2 aligns with, diverges from, or complicates current models of viral latency, immune control, and reservoir persistence, we aim to distinguish robust observations from those that remain speculative or context-dependent.

This review examines key aspects of HIV-2 molecular biology, including interactions with host restriction factors, innate and adaptive immune responses, and the dynamics of latent reservoirs. Evidence from the literature is considered alongside conflicting findings where they exist. By taking this evidence-weighted perspective, we explore how insights from HIV-2 may inform HIV-1 remission research, not as definitive solutions, but as a way to identify biological constraints and unresolved questions that any durable cure strategy must ultimately address.

## 2 Molecular and virological features of HIV-2

**Figure 1:**
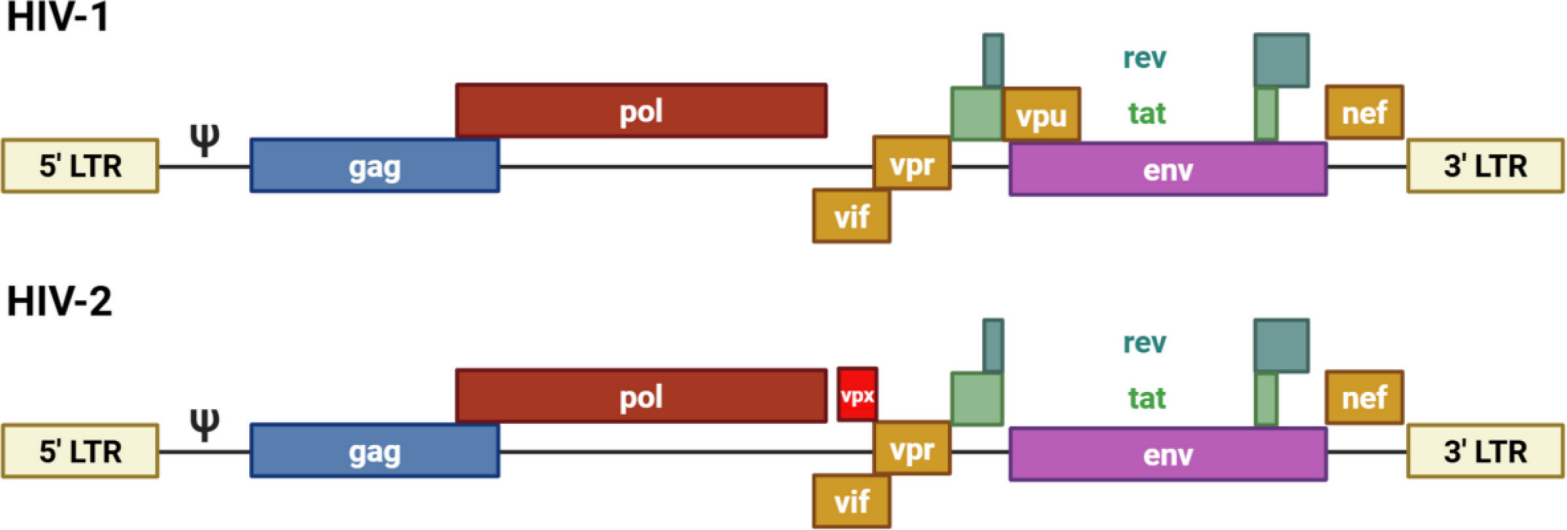
Genome organization of HIV-1 and HIV-2. Schematic representation of the HIV-1 and HIV-2 genomes shown in the 5′→3′ orientation, including major structural genes (gag, pol, env), regulatory genes (tat, rev, nef), and selected accessory genes. HIV-1 encodes vpu, whereas HIV-2 encodes vpx, which antagonizes the host restriction factor SAMHD1. Long terminal repeats (LTRs) and the packaging signal (Ψ) are indicated for both viruses.

HIV-2 shares the same overall genomic organization as HIV-1, including the canonical gag, pol, and env genes, flanked by long terminal repeats (LTRs), and encodes multiple accessory proteins that modulate host–virus interactions. **Figure 1** provides an overview of the genomic organization of HIV-1 and HIV-2, highlighting shared structural features and selected differences in accessory gene content that have been experimentally described (11).

However, important distinctions in genome content, protein function, and replication dynamics distinguish HIV-2 from HIV-1 and have been proposed to influence viral persistence and pathogenesis (1).

**Table 1** summarizes selected molecular and genomic features that distinguish HIV-2 from HIV-1, as reported across multiple experimental studies.

**Table 1:** Selected molecular differences between HIV-2 and HIV-1.

Feature	HIV-2	HIV-1	References
Accessory proteins	Encodes Vpx (SAMHD1 degradation in myeloid cells and resting T cells); lacks Vpu	Encodes Vpu (tetherin antagonism via proteasomal/endosomal routing); lacks Vpx	(12, 13, 15)
SAMHD1 antagonism	Efficient Vpx-mediated proteasomal degradation via CRL4(DCAF1) E3 ubiquitin ligase; enhances infection of macrophages and dendritic cells	None; SAMHD1 restricts HIV-1 replication in myeloid cells and resting CD4+ T cells by depleting dNTP pools	(12, 13, 57)
Tetherin antagonism	Partial Env-mediated counteraction; mechanism distinct from Vpu; variable across primary isolates	Robust Vpu-mediated antagonism via AP-1-dependent exclusion from virus assembly sites and proteasomal/lysosomal degradation	(15–18)
Tat activity	Structural divergence from HIV-1 Tat (<30% sequence homology); LTR contains duplicated TAR RNA stem-loops; Y44A substitution in acidic domain markedly reduces LTR-transactivation; subtype-dependent differences in transcriptional efficiency *in vitro*	Robust Tat-mediated transcriptional elongation via P-TEFb/CycT1 recruitment to single TAR stem-loop; well-characterised stimulatory effects *in vivo*	(24, 49, 51, 58)
Nef functions	Structurally distinct from HIV-1 Nef: extra C-terminal helix; altered AP binding affinity; variable modulation of CD4 and MHC-I surface expression; reduced pathogenic potency compared to HIV-1 Nef	Efficient CD4 downregulation via AP-2 hijacking (dileucine motif); strong MHC-I internalization and sequestration via beta-COP pathway; major pathogenesis determinant	(59–61)
Env binding & coreceptor usage	*In vitro* evidence of broader coreceptor usage (CCR5, CXCR4, CCR8, and others); context-dependent entry efficiency; *in vivo* relevance of expanded tropism still under investigation	Primarily CCR5 (early/chronic infection) and CXCR4 (late/CXCR4-tropic variants); efficient CD4 binding; well-characterised tropism switch	(21–23, 25)

One defining molecular difference between the two viruses is the presence of the accessory protein Vpx in HIV-2 and its absence in HIV-1. Vpx antagonizes the host restriction factor SAMHD1, thereby facilitating reverse transcription in myeloid cells and resting CD4^+^ T cells (12, 13). In contrast, HIV-1 lacks Vpx and instead encodes Vpu, which counteracts tetherin to enhance virion release (14–16). HIV-2 does not encode Vpu; rather, its Env protein has been shown to mediate tetherin antagonism (14, 15, 17, 18). These differences reflect alternative strategies of host restriction factor antagonism rather than a demonstrated evolutionary trade-off between these functions.

Comparative structural studies have revealed differences in the organization of the HIV-2 capsid lattice relative to HIV-1, as determined by cryo-electron microscopy and biochemical analyses (19). While these studies demonstrate architectural variation, direct experimental evidence linking HIV-2 capsid structure to altered innate immune sensing or differential activation of cytosolic DNA sensors such as cGAS or IFI16 remains limited. As such, any impact of capsid architecture on innate immune activation during HIV-2 infection should be regarded as a hypothesis rather than a demonstrated mechanism (1, 20).

*In vitro* studies suggest that some HIV-2 Env variants may use a broader array of coreceptors and exhibit context-dependent differences in cell entry (21–23). Earlier studies proposed that HIV-2 Env exhibits systematically lower CD4 binding affinity than HIV-1 Env (23), but subsequent analyses have demonstrated that observed differences in entry efficiency are largely strain-specific or context-dependent rather than a consistent feature across primary HIV-2 isolates (21, 22). The *in vivo* relevance of any systematic differences in CD4 affinity, therefore, remains to be fully established.

At the level of transcriptional regulation, sequence divergence within the HIV-2 LTR and functional variation in Tat-mediated transactivation have been described (1, 11, 24). These features have been proposed to influence viral gene expression dynamics. Importantly, such molecular differences do not, on their own, establish that HIV-2 transcription is intrinsically attenuated relative to HIV-1, particularly in the absence of direct *in vivo* comparative measurements.

## 3 Immune modulation and control mechanisms

HIV-2 infection is frequently characterized by delayed disease progression and preserved immune competence, particularly during early and intermediate stages of infection (1, 3, 25). These clinical features have motivated extensive investigation into how HIV-2 modulates host immune responses and how such modulation may differ from HIV-1 infection.

### 3.1 Innate immune activation and inflammatory responses

Early studies suggested that HIV-2 infection may be associated with reduced innate immune activation compared with HIV-1. However, subsequent investigations have demonstrated that monocyte and myeloid dendritic cell activation occurs throughout HIV-2 infection, including in individuals with low or undetectable plasma viral loads (26), the same aviremic individuals in whom cell-associated viral RNA has been detected, indicating ongoing viral replication despite undetectable plasma viremia (9). Notably, CD8^+^ T-cell activation persists even in aviremic individuals, albeit at low levels, whereas CD4^+^ T-cell activation was not statistically significant in the aviremic group, a distinction that reflects selective, targeted immune engagement rather than global immune activation (26). Moreover, inflammatory markers in HIV-2 infection correlate positively with viral load and, when normalized for viremia, may exceed levels observed in HIV-1 infection (26).

The role of SAMHD1 in shaping innate immune responses further complicates this picture. Beyond restricting viral replication, SAMHD1 has been shown to restrain baseline and virus-induced inflammatory signalling in myeloid cells, including through suppression of the MAVS–IKKϵ–IRF7 interferon axis and inhibition of NF-κB and interferon pathways (27–29). Thus, while Vpx-mediated antagonism of SAMHD1 enhances HIV-2 replication in certain cellular contexts, it may also disinhibit innate immune signalling in ways that do not uniformly favour immune quiescence, a possibility supported by evidence that HIV-2/SIV Vpx can itself modulate NF-κB activation (5). This represents a biologically plausible hypothesis that warrants further *in vivo* validation before it can be considered a settled mechanism.

Together, these findings indicate that HIV-2 infection does not universally evade innate immune sensing but instead induces a regulated inflammatory response that varies with viral burden and disease stage.

### 3.2 Adaptive immune responses: T cells, antibodies, and NK cells

Preservation of CD4^+^ T-cell counts and sustained functional CD8^+^ T-cell responses have been documented in many individuals with HIV-2 infection, particularly during prolonged asymptomatic phases (1, 3, 30).

HIV-2–specific cytotoxic T lymphocytes have been shown to maintain polyfunctionality and proliferative capacity, which may contribute to long-term viral containment (1, 30, 31). It should be noted, however, that direct comparative studies of HIV-2–specific CTL polyfunctionality using standardised assays remain limited, and the degree to which these features are quantitatively superior to those observed in HIV-1 infection has not been systematically established.

Humoral responses in HIV-2 infection have been reported, in some studies, to show broad and potent neutralizing activity; these features vary among individuals and do not imply uniform superiority relative to HIV-1 (32–34). In addition, antibody-dependent cellular cytotoxicity (ADCC) has been proposed to contribute to immune-mediated control of infected cells. While direct studies of ADCC in HIV-2 infection are limited, cross-reactive antibodies with the capacity to mediate HIV-1 envelope-targeted ADCC have been identified in HIV-2–infected individuals (35), suggesting that similar mechanisms may contribute to control in HIV-2.

Natural killer (NK) cell activity has also been implicated in immune control during HIV infection. In HIV-1 infection, high viremia is associated with expansion of dysfunctional CD56^-^/CD16^+^ NK cell subsets and impaired cytotoxic receptor signalling, while viral controllers tend to maintain activated effector NK cell phenotypes with favourable receptor expression profiles (36–38). Direct comparative data on NK cell function specifically in HIV-2 infection remain sparse; available evidence should therefore be interpreted cautiously, and the assumption that NK cell profiles in HIV-2 are uniformly more favourable than in HIV-1 is not yet supported by systematic comparative studies. As with other immune parameters, NK cell features are heterogeneous across individuals and should not be interpreted as universally protective.

### 3.3 Integrated immune control rather than immune silence

Collectively, available data support a model in which HIV-2 persistence is shaped by active immune surveillance rather than immune evasion or global immune quiescence. Both innate and adaptive immune responses remain engaged throughout infection, with their magnitude and effectiveness closely linked to viral replication levels. The degree to which these immune features are quantitatively or qualitatively distinct from those in HIV-1 infection remains an open question that requires direct comparative investigation using standardised cohorts and assay platforms.

It is important to note that HIV-1 infection also exhibits substantial individual-level heterogeneity in viral load, CD4^+^ T-cell trajectory, immune activation, and reservoir size across patients, populations, and geographies, yet the HIV-1 field has successfully extracted generalizable, population-level insights from this variability. The same principle applies to HIV-2. The comparative value of HIV-2 as a biological system does not depend on uniformity across all infected individuals; rather, it lies in the directional consistency of certain biological features, lower median plasma viremia, slower average CD4^+^ T-cell decline, and preserved immune function in a larger proportion of individuals that are reproducible across independent cohorts and geographies despite individual variation (1–4). These directional differences, even against a background of heterogeneity, provide a meaningful biological signal for HIV-1 cure research.

## 4 Latency, reservoir dynamics, and translational relevance

A central question in HIV cure research is whether differences in viral biology or host immune control translate into qualitative or quantitative differences in the establishment, maintenance, or inducibility of latent viral reservoirs. HIV-2 infection has attracted attention in this context due to its association with delayed disease progression and prolonged periods of low-level viremia in some individuals (1, 39). These observations have prompted an investigation into whether HIV-2 reservoirs differ fundamentally from those observed in HIV-1 infection.

**Table 2** summarises representative reservoir-related measurements reported for HIV-1 and HIV-2 infection across different cohorts and assay platforms.

**Table 2:** Comparative reservoir characteristics of HIV-1 and HIV-2.

Feature	HIV-1	HIV-2	References
Estimated intact proviral frequency	~50–300 per 10^6 CD4+ T cells (assay-dependent; IPDA and QVOA estimates vary)	Detectable but variably reported; cross-comparison limited by assay differences and absence of validated HIV-2-specific IPDA	(41, 62, 63)
Fraction of inducible proviruses	Typically <5-20% by QVOA; higher with strong stimulation; low inducibility is a major barrier to cure	Inducible virus detected *in vitro*; quantitative comparisons with HIV-1 limited; HIV-2 shows reduced reactivation sensitivity relative to HIV-1 in reporter virus models	(53, 64)
Proviral integrity and structure	Mixture of intact and defective proviruses; defective proviruses outnumber intact by >12-fold; clonal expansion of infected cells common	Proviral landscape dominated by defective proviruses; clonal expansion observed in some cohorts; intact proviral frequency lower than HIV-1 in available data	(40, 41, 62)
Integration-site characteristics	Strong enrichment in actively transcribed genes, gene-dense chromatin, and speckle-associated domains; mediated by LEDGF/p75-integrase interaction and CPSF6-capsid interaction	Limited comparative data; no consistent evidence for fundamentally distinct integration targeting relative to HIV-1; LEDGF/p75 interaction conserved	(65–68)
Immune activation during chronic infection	Persistent immune activation despite ART; elevated inflammatory markers; contributes to non-AIDS morbidity	Variable; generally lower than HIV-1; correlates with viral load and disease stage; aviremic HIV-2 patients show evidence of ongoing low-level replication	(9, 31, 69)
Estimated reservoir stability / half-life	~3–4 years (t1/2) for intact proviruses in ART-suppressed infection; biphasic decay pattern; stabilisation after ~7–10 years of ART	Unknown; insufficient longitudinal data for reliable estimation; no validated assay for HIV-2 intact proviral DNA decay kinetics	(70–72)

### 4.1 Establishment and composition of the HIV-2 proviral reservoir

Integrated HIV-2 proviruses can be readily detected in peripheral blood mononuclear cells from infected individuals, including those with long-standing infection and low plasma viral loads (9). Molecular analyses have demonstrated that HIV-2 proviruses exhibit heterogeneous structures, including intact and defective genomes, as well as evidence of clonal expansion within infected cell populations (40–42).

Importantly, some comparative analyses have noted that aspects of the HIV-2 proviral landscape can resemble those observed in HIV-1, but this conclusion is based on limited cohort data and warrants further study (41). These findings challenge interpretations advanced in prior reviews and comparative studies, including proposals that HIV-2 may serve as a naturally occurring model of functional cure due to a presumptively smaller or more controlled reservoir (31, 43), that HIV-2 reservoirs are intrinsically small, structurally inert, or transcriptionally silenced. Rather, reservoir composition appears to reflect common processes of integration, cellular proliferation, and immune-mediated selection that operate across both HIV types (1, 31, 40).

### 4.2 Transcriptional activity and inducibility of HIV-2 proviruses

Several studies have examined HIV-2 transcriptional activity *in vivo* by measuring cell-associated viral RNA. Quantification of unspliced gag RNA in CD4-matched individuals infected with HIV-1 or HIV-2 revealed comparable levels of viral transcription in peripheral blood cells when normalised for CD4^+^ T-cell counts and disease stage (9). These data indicate that HIV-2 proviruses are transcriptionally active *in vivo* and challenge the view that HIV-2 is characterised by globally attenuated transcription. However, it should be noted that other studies have reported lower per-cell viral transcriptional output in HIV-2 compared with HIV-1 (44). The weight of evidence therefore suggests that HIV-2 does not maintain a uniformly silenced transcriptional programme, but the degree of transcriptional restriction relative to HIV-1 may vary with disease stage, cohort composition, and measurement methodology.

Experimental latency models using molecularly cloned HIV-2 reporter viruses have demonstrated that HIV-2 proviruses can enter transcriptionally silent states and can be reactivated under appropriate stimulation (40, 41). However, such *in vitro* systems may not fully capture the complexity of latency and inducibility in infected individuals and should be interpreted cautiously when extrapolated to *in vivo* reservoir dynamics.

Taken together, available evidence supports the conclusion that HIV-2 latency reflects regulated transcriptional activity rather than permanent or irreversible proviral silencing.

### 4.3 Reservoir size: evidence, uncertainty, and methodological constraints

Several epidemiological and clinical studies have reported lower plasma viral loads in HIV-2 infection compared with HIV-1, particularly during early and intermediate disease stages (1, 2). These observations have sometimes been extrapolated to suggest that HIV-2 reservoirs are smaller or less inducible.

Direct quantitative comparisons of reservoir size between HIV-1 and HIV-2 infection, however, remain limited and methodologically challenging. Differences in assay sensitivity and primer/probe compatibility, compounded by viral sequence diversity, complicate cross-species measurements of proviral DNA and inducible virus. In addition, many studies rely on peripheral blood sampling, which may not reflect reservoir dynamics in lymphoid tissues or other anatomical compartments (1, 39, 40).

Notably, in a detailed molecular analysis, Koofhethile and co (41) concluded that the HIV-2 proviral landscape is dominated by defective proviruses, a composition closely resembling that observed in HIV-1 infection, underscoring the lack of definitive evidence for a uniquely small or transcriptionally inert HIV-2 reservoir. Accordingly, claims of reduced reservoir size in HIV-2 infection should be regarded as provisional and context-dependent, given methodological challenges and limited cross-species comparative data.

### 4.4 Immune pressure and reservoir maintenance

The maintenance of latent reservoirs is shaped not only by viral factors but also by host immune responses. Sustained CD8^+^ T-cell activity, NK cell function, and antibody-mediated responses observed in many individuals with HIV-2 infection (30, 31, 45) may contribute to limiting the expansion of transcriptionally active infected cells.

This model emphasises immune-mediated containment rather than intrinsic viral quiescence as a key determinant of reservoir dynamics. Under such conditions, cells expressing higher levels of viral antigen may be preferentially cleared, while cells with lower or intermittent expression persist. This selective pressure could contribute to the long-term stability of the reservoir without necessitating profound transcriptional repression, despite ongoing low-level transcription.

### 4.5 Translational implications and limitations

HIV-2 infection has been proposed as a naturally occurring analogue of functional remission based on its association with delayed disease progression and prolonged viral control in some individuals. However, the evidence reviewed here indicates that HIV-2 persistence is compatible with ongoing viral transcription, reservoir maintenance, and eventual disease progression in a substantial proportion of cases (1, 2, 31, 40).

As such, HIV-2 should not be viewed as a model of durable proviral silencing or reservoir eradication. Instead, its relevance to cure research lies in illustrating how immune surveillance and regulated viral expression can coexist over extended periods. These observations inform, but also constrain, the design of HIV-1 remission strategies by highlighting the limitations of approaches that focus exclusively on transcriptional suppression without addressing reservoir persistence and immune engagement (1, 40).

An important contextual consideration is that HIV-2 infection is geographically concentrated in West Africa, a region characterised by distinct host genetic profiles and a high burden of endemic co-infections. West African populations harbour diverse human leukocyte antigen (HLA) allele frequencies and carry variants of innate immune restriction factors, including tripartite motif-containing protein 5α (TRIM5α), that have been associated with differential retroviral control (25). In addition, endemic co-infections prevalent in this region, including malaria, helminthiasis, and tuberculosis, are known to modulate immune activation, CD4^+^ T-cell homeostasis, and inflammatory tone in ways that could independently influence HIV-2 disease trajectory (46). These host and environmental factors represent important confounders that limit the extent to which the HIV-2 phenotype can be attributed exclusively to viral biology, and they underscore the need for geographically diverse cohort studies when drawing generalizable conclusions about HIV-2 immunopathogenesis.

## 5 Viral transcriptional regulation in HIV-2

Transcriptional regulation is a central determinant of HIV replication, latency establishment, and reservoir maintenance. In both HIV-1 and HIV-2, productive infection depends on host transcription factors and the viral transactivator Tat, which enhances RNA polymerase II processivity through interactions with the transactivation response (TAR) element in the viral LTR (24, 47, 48). Differences in LTR architecture and Tat sequence between HIV-1 and HIV-2 have therefore attracted attention as potential contributors to differences in replication dynamics and clinical outcomes.

### 5.1 LTR architecture and Tat–TAR interactions

The HIV-2 LTR differs structurally from that of HIV-1, most notably in the presence of duplicated TAR elements, two tandem stem-loop structures in contrast to the single stem-loop of HIV-1 TAR, and in variation in transcription factor binding sites within the U3 region (1, 11, 24). The duplicated TAR structure of HIV-2 is required for efficient Tat-mediated transactivation, with the proximal stem-loop playing a dominant role in transcriptional elongation (24, 49).

Comparative analyses of HIV-2 group A and group B LTRs have demonstrated high genetic variability within the U3 subregion, which encompasses most known transcription factor binding sites, and group-specific differences in basal and Tat-responsive transcriptional activity. These findings indicate that regulatory heterogeneity within HIV-2 may contribute to differences in proviral reservoir size between groups (11, 50). LTR-driven transcription in HIV-2 is therefore context-dependent and modulated by viral genetic variation.

HIV-2 Tat also exhibits sequence divergence relative to HIV-1 Tat, including differences within the acidic domain and regions implicated in transcriptional elongation. A mutational analysis identified a Y44A substitution in the acidic domain of HIV-2 Tat that markedly reduced LTR-mediated transactivation and viral reverse transcriptase expression *in vitro* (11, 51). This study demonstrates that specific HIV-2 Tat sequence variants influence transactivation *in vitro*; however, it does not directly compare HIV-2 and HIV-1 transcriptional outputs during primary infection.

### 5.2 Transcriptional activity of HIV-2 in natural infection

Low plasma viral loads and delayed disease progression observed in many individuals with HIV-2 infection (1, 52) have prompted investigation into whether viral gene expression differs fundamentally between HIV-2 and HIV-1 *in vivo*. However, this assumption has largely been inferred from virological endpoints rather than demonstrated through direct comparative measurements of transcriptional activity.

Direct *in vivo* evidence (44) provides important context for evaluating claims of globally attenuated HIV-2 transcription, though the picture is not uniform across studies. Quantification of unspliced gag RNA in peripheral blood mononuclear cells from CD4-matched individuals infected with HIV-1 or HIV-2 revealed comparable levels of cell-associated viral RNA, indicating similar steady-state transcriptional activity between the two viruses (9). In contrast, MacNeil et al. (44) reported significantly lower per-cell HIV-2 transcriptional output relative to HIV-1, suggesting that the degree of transcriptional restriction may vary with cohort composition and measurement approach (44). Taken together, these data argue against transcription initiation or elongation as the sole determinant of the lower viral loads characteristic of HIV-2 infection, while acknowledging that some degree of transcriptional restriction may exist in certain clinical contexts.

Rather, the attenuated replication phenotype of HIV-2 appears to reflect post-transcriptional and translational constraints. Differences in virion production efficiency and protein expression levels, mechanisms mechanistically downstream of transcription, have been documented in HIV-2 and may contribute to reduced plasma viraemia without implying reduced transcriptional output per se (11). Accurate interpretation of HIV-2 pathobiology, therefore, requires careful distinction between transcriptional regulation and downstream steps in the viral gene expression cascade.

### 5.3 Transcriptional regulation and latency phenotypes

Latency is increasingly recognised as a spectrum of transcriptional states rather than a binary on–off phenomenon (39). In this context, differences between HIV-1 and HIV-2 may reside not in absolute transcriptional capacity, but in the responsiveness, kinetics, or regulatory stability of proviral expression. This possibility is consistent with observed heterogeneity in LTR activity across HIV-2 groups and sensitivity of Tat-mediated transactivation to sequence variation (11, 39, 50).

Claims that HIV-2 latency is characterised by reduced inducibility or fundamentally weaker transcriptional programmes have largely been derived from *in vitro* systems employing reporter viruses or molecular clones (49). Such models are informative but provide limited support for generalised conclusions about HIV-2 latency *in vivo*, given the complexity of transcriptional regulation and immune interactions. Notably, *in vitro* comparisons using a dual-reporter HIV-2 system demonstrated that HIV-2 is less cytotoxic and less sensitive to latency reactivation than HIV-1 under cell culture conditions, though the *in vivo* relevance of these findings requires further investigation (53).

Taken together, available evidence indicates that HIV-2 transcriptional regulation differs from HIV-1 in architecture and regulatory sensitivity, but does not support broad assertions that HIV-2 proviruses are intrinsically transcriptionally attenuated in infected individuals (9, 11). Instead, HIV-2 highlights the need to refine latency models to incorporate regulatory nuance, viral heterogeneity, and the integration of transcriptional activity with immune-mediated clearance. These distinctions are relevant for HIV-1 cure research, but should be interpreted as hypothesis-generating rather than prescriptive for ‘block-and-lock’ strategies (39, 47).

## 6 HIV-2 infection as a comparative model for functional HIV-1 remission

### 6.1 Clinical and epidemiological features relevant to functional remission

Interest in HIV-2 as a comparator for functional HIV-1 remission has been driven by its distinct epidemiological and clinical features, including lower plasma viral loads, delayed disease progression, and preserved immune competence in a subset of infected individuals (1, 2). These characteristics overlap conceptually with the goals of remission-oriented cure strategies, which aim to maintain long-term viral suppression without continuous antiretroviral therapy (31).

However, clinical similarity alone does not establish mechanistic equivalence. While HIV-2 infection has often been associated with prolonged periods of viral control, longitudinal cohort studies demonstrate that a substantial proportion of untreated individuals ultimately experience CD4^+^ T-cell decline and progression to AIDS (2, 43). These findings indicate that HIV-2 persistence reflects delayed pathogenesis rather than spontaneous viral eradication or permanent remission.

### 6.2 Transcriptional regulation and viral gene expression in HIV-2

As detailed in Section 5, the available evidence does not support a model of globally attenuated HIV-2 transcription, though the degree of transcriptional restriction relative to HIV-1 remains debated (9, 44). The key translational implication is that transcription-targeting interventions, including Tat inhibitors and epigenetic silencing agents, are unlikely to achieve durable remission in HIV-1 if applied in isolation, because the HIV-2 comparator demonstrates that ongoing transcription can coexist with prolonged immune containment. Effective HIV-1 cure strategies will therefore need to address both transcriptional regulation and the immune mechanisms that constrain transcriptionally active reservoirs (11, 39, 47).

### 6.3 Latent reservoir persistence and immune containment

As detailed in Sections 3 and 4, the HIV-2 proviral landscape closely resembles that of HIV-1 in its composition of defective and intact proviruses and its evidence of clonal expansion (41, 43). The translational implication is that reservoir persistence in HIV-2 is maintained by active immune surveillance rather than intrinsic viral quiescence, a finding that directly challenges the premise that transcriptional silencing alone is sufficient for durable remission. For HIV-1 cure research, this underscores the importance of strategies that preserve or enhance antiviral immune responses alongside any transcription-targeting intervention (1, 31, 40).

### 6.4 Relevance to remission-oriented therapeutic strategies

Block-and-lock approaches aim to enforce stable transcriptional repression of integrated proviruses to prevent viral rebound (54, 55). HIV-2 has occasionally been cited as a naturally occurring analogue of remission-like states, but the evidence indicates that persistence coexists with ongoing transcription and eventual progression in many cases (1, 31).

The evidence reviewed here does not support HIV-2 as a model of complete or irreversible transcriptional silencing. Rather, HIV-2 illustrates a state in which regulated viral expression coexists with effective immune containment. For HIV-1, this suggests that transcription-targeting interventions, such as Tat inhibition, epigenetic modulation, or capsid-directed strategies, are unlikely to be sufficient in isolation and may need to be combined with approaches that preserve or enhance antiviral immune responses (11, 39).

### 6.5 Translational considerations and limitations

The principal contribution of HIV-2 to cure research lies in defining biological constraints. HIV-2 demonstrates that long-term viral persistence can occur alongside immune competence and regulated transcription, without requiring complete proviral silence or reservoir elimination (1, 41).

Accordingly, HIV-2 should be viewed as a comparative system that informs the limits of transcription-centred cure paradigms and underscores the importance of multifactorial strategies. Its study highlights the need to integrate virological control with sustained immune surveillance when designing approaches aimed at durable HIV-1 remission.

## 7 Conclusion

HIV-2 infection offers a unique comparative context in which viral persistence, immune competence, and delayed disease progression can coexist. While early clinical observations led to the perception of HIV-2 as a naturally attenuated virus, accumulating molecular and epidemiological data demonstrate substantial heterogeneity in transcriptional activity, reservoir composition, and clinical outcome across infected individuals (11, 24, 39, 41, 43, 47, 56).

Direct comparisons to date do not indicate that HIV-2 is characterized by globally reduced viral transcription *in vivo*; evidence varies with cohort and measurement methods (9), nor by a uniformly small or inert latent reservoir, given existing methodological and cohort limitations.

Rather than providing a prescriptive model for cure, HIV-2 highlights the complexity of achieving durable viral control in the absence of therapy. Its study underscores the importance of immune surveillance, regulatory nuance in viral gene expression, and host–virus equilibrium in shaping long-term outcomes. For HIV-1 cure research, the principal contribution of HIV-2 lies in refining expectations, challenging over-simplified paradigms, and informing the design of multifactorial strategies that integrate transcriptional modulation with sustained immune engagement.
